# Crystalline Structure, Defect Chemistry and Room Temperature Colossal Permittivity of Nd-doped Barium Titanate

**DOI:** 10.1038/srep42274

**Published:** 2017-02-13

**Authors:** Qiaomei Sun, Qilin Gu, Kongjun Zhu, Rongying Jin, Jinsong Liu, Jing Wang, Jinhao Qiu

**Affiliations:** 1 State Key Laboratory of Mechanics and Control of Mechanical Structures, College of Aerospace Engineering, Nanjing University of Aeronautics and Astronautics, Nanjing 210016, China; 2 College of Materials Science and Technology, Nanjing University of Aeronautics and Astronautics, Nanjing 210016, China; 3 Department of Physics and Astronomy, Louisiana State University, Baton Rouge, LA 70803, USA

## Abstract

Dielectric materials with high permittivity are strongly demanded for various technological applications. While polarization inherently exists in ferroelectric barium titanate (BaTiO_3_), its high permittivity can only be achieved by chemical and/or structural modification. Here, we report the room-temperature colossal permittivity (~760,000) obtained in *x*Nd: BaTiO_3_ (*x* = 0.5 mol%) ceramics derived from the counterpart nanoparticles followed by conventional pressureless sintering process. Through the systematic analysis of chemical composition, crystalline structure and defect chemistry, the substitution mechanism involving the occupation of Nd^3+^ in Ba^2+^ -site associated with the generation of Ba vacancies and oxygen vacancies for charge compensation has been firstly demonstrated. The present study serves as a precedent and fundamental step toward further improvement of the permittivity of BaTiO_3_-based ceramics.

The development of high-energy-density storage devices is extremely urgent for the sake of advanced microelectronics and communications^1^,^2^,^3^. Of great importance is to search for appropriate dielectric materials with high permittivity^4^,^5^. Inorganic ceramic materials with high dielectric constant have received extensive attention, due to their application in multilayer ceramic capacitors (MLCC)^6^ and inorganic-organic hybrid flexible composite films^7^. The dielectric properties of inorganic ceramic materials can be improved *via* chemical modification^8^,^9^ and/or grain-size engineering^10^. Among them, rare-earth element(s) doped BaTiO_3_ (RE: BaTiO_3_) has been considered as one of the most suitable materials for ferroelectric capacitors, because of its colossal dielectric constant (CDC)^11^. The incorporation of trivalent RE ions (such as La^3+^ and Nd^3+^) can effectively enhance the room temperature permittivity of BaTiO_3_^12^,^13^, thereby improving the performance of relevant energy storage devices. In addition, the dielectric properties of BaTiO_3_ can be tuned by varying the grain size; that is, with the decreasing grain size, the permittivity initially increases and then decreases after reaching a maximum at a critical grain size^10^.

The permittivity of RE: BaTiO_3_ is intimately related to the structural distortion and chemical defects surrounding the dopants. Theoretical calculations in terms of tolerance factor have indicated that the occupation of the exotic ions depends on their radius^14^,^15^. Since the radius of RE ions is usually between Ba^2+^(1.35 Å) and Ti^4+^(0.68 Å), larger RE^3+^ ions such as La^3+^ (1.15 Å) and Nd^3+^(1.08 Å) prefer to substitute for the Ba^2+^ -site (A-site), and the smaller ones such as Yb^3+^(0.87 Å) may locate exclusively at the Ti^4+^ -site (B-site), while the intermediate ones such as Y^3+^(0.93 Å) and Er^3+^(0.96 Å) may occupy both the A- and B- sites^16^,^17^. Accompanying with the specific doping, chemical inhomogeneity is often introduced. For example, the partial replacement of the A-site by La^3+^would give rise to either the formation of Ti vacancies or the reduction of Ti^18^. These defects could significantly affect the dielectric properties of BaTiO_3_-based ceramics. As demonstrated by Guillemet-Fritsch *et al*.^19^, a room-temperature colossal permittivity (*ε*_eff_ ~800,000) was obtained in Ba_0.95_La_0.05_TiO_3-*x*_ ceramics sintered by the spark plasma sintering (SPS) method, in which Ti^3+^/Ti^4+^ acted as polaron carriers.

Although Nd: BaTiO_3_ ceramics have been studied^13^,^20^,^21^,^22^, the mechanism for their improved permittivity (~300,000) remains unclear^13^. In addition, Nd: BaTiO_3_ powders in the previous reports are often prepared by solid-state reaction (SSR), and high-temperature treatment (above 800 °C) is usually required, which results in large grain size and poor sintering activity. To achieve high bulk density and improved dispersion in organic matrix, ultrafine Nd: BaTiO_3_ nanoparticles with pure phase and uniform particle size are highly desirable yet challenging. Although it is well known that hydrothermal synthesis can yield high-purity nanoparticles with narrow size distribution^23^,^24^, the synthesis of RE: BaTiO_3_ nanoparticles has been rarely reported^25^,^26^. Recently, we have synthesized monodispersed BaTiO_3_ nanoparticles *via* the sol-hydrothermal method^27^. In this work, *x*Nd: BaTiO_3_ (*x* = 0 ~3 mol%) nanocrystals were prepared by a similar process and their crystalline structure and defect chemistry were investigated elaborately. Subsequently, *x*Nd: BaTiO_3_ ceramics were fabricated by a conventional pressureless sintering method. It’s found that the dielectric constant was dramatically modified upon Nd doping. Especially, the colossal dielectric constant was observed in the sample with 0.5 mol% Nd at room temperature, and possible mechanisms for such effect were provided.

## Results and Discussion

### Crystalline structure analysis of *x*Nd: BaTiO_3_ nanocrystals

**Figure 1:**
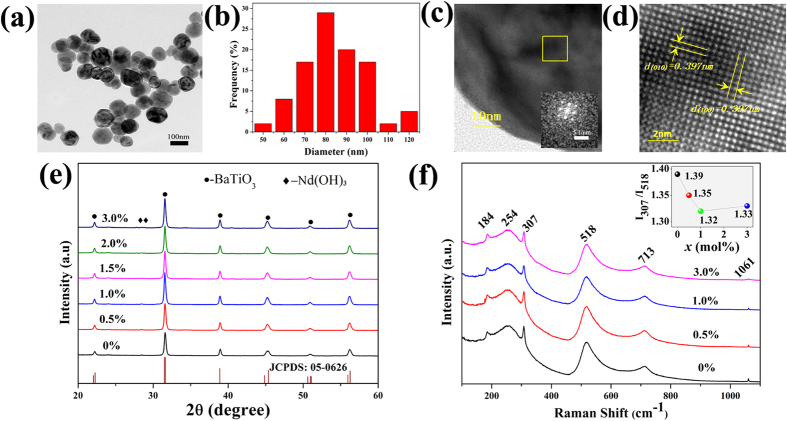
(**a**) TEM image of pure BaTiO_3_ nanoparticles; (**b**) Particle size distribution of pure BaTiO_3_ nanoparticles; (**c**) TEM image of an individual particle and its fast Fourier transform pattern in the inset; (**d**) HR-TEM image of the selected area marked by the yellow square in (**c**); (**e**) XRD patterns of *x*Nd: BaTiO_3_ nanoparticles with indication of nominal Nd concentration; (**f**) Raman spectra of *x*Nd: BaTiO_3_ samples with the inset showing the Nd-doping dependence of the relative intensity of peaks at 307 and 518 cm^−1^.

The as-prepared *x*Nd: BaTiO_3_ nanocrystals were characterized by various techniques. Figure 1a shows the transmission electron microscopy (TEM) image of pure BaTiO_3_. The size distribution of these spherical particles is displayed in Fig. 1b, indicating that the diameter of majority particles (~90%) is in the range of 60~100 nm. Zooming into an individual nanoparticle (Fig. 1c), a single crystal character is revealed, as demonstrated by the fast Fourier transform (FFT) pattern (the inset of Fig. 1c) and well-defined atomic arrangement in the HR-TEM image in Fig. 1d. The interplanar spacing of the lattice is 0.397 nm, corresponding to (100) plane in tetragonal BaTiO_3_.

The tetragonal structure of nanoparticles has been further confirmed by X-ray diffraction (XRD) patterns. As shown in Fig. 1e, all XRD peaks obtained from pure BaTiO_3_ nanoparticles are well-matched with the tetragonal structure corresponding to Joint Committee on Powder Diffraction Standards (JCPDS) Files No. 05-0626. These peaks remain in the *x*Nd: BaTiO_3_ nanocrystals (see Fig. 1e). Inductively coupled plasma optical emission spectrometry (ICP-OES) was further carried out to quantify the chemical composition of samples. As listed in Table 1, the actual Nd concentration is found to be 0, 0.49, 0.95 and 1.43 mol% for nominal *x*Nd: BaTiO_3_ samples with *x* = 0, 0.5, 1.0 and 1.5 mol%, respectively. The result indicates that the Nd ions have almost completely incorporated into the BaTiO_3_ structure.

However, additional peaks (as indicated by diamond symbol) appear in the sample with *x* = 2 mol%, which can be assigned to Nd(OH)_3_ impurity phase (JCPDS No. 06-0601). Also, the energy dispersive X-ray spectroscopy (EDX) analysis results in Supplementary Fig. S2 confirm that rod-like impurity can be attributed to Nd(OH)_3_.

To confirm that the BaTiO_3_ nanocrystals form only tetragonal structure at room temperature, we further measured Raman vibrational spectroscopy, as it is sensitive to the structure symmetry^1^,^28^. As can be seen from Fig. 1f, all Raman scattering spectra consist of bands around 184, 254, 307, 518 and 713 cm^−1^, which are the characters of perovskite BaTiO_3_. While the small peak at 1061 cm^−1^ denotes the presence of BaCO_3_ and the intensity decreases with the doping concentration. It is well-known that all phonons of cubic P*m*
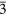
*m* symmetry are inactive to Raman modes, due to the isotropic distribution of electrostatic forces. The tetragonal P4*mm* space group exhibit eight Raman active modes described by 3[A_1_(TO) + A_1_(LO)] + 4[E(TO) + E(LO)] + B_1_. Among these, peaks at 184, 254, 518 cm^−1^ are assigned to the fundamental TO mode of A_1_ symmetry, which generally exist both in cubic and tetragonal BaTiO_3_^29^. The presence of bands at 184 cm^−1^ indicates a decoupling between the A_1_(TO) phonons, which can be induced by internal stress or lattice defects; whereas the asymmetry in the bands at 518 cm^−1^ suggests the existence of coupling of the TO modes associated with the tetragonal phase. The sharp peak at 307 cm^−1^ corresponding to [B_1_, E(TO + LO)] modes is attributed to the non-centrosymmetric regions arising from the displacement of titanium atoms from TiO_6_ octahedra^30^,^31^, suggesting the intrinsic structural distortion in the tetragonal BaTiO_3_. The appearance of peak at 713 cm^−1^ is considered to the highest frequency longitudinal optical mode (LO). Besides, the higher relative intensity of the band *versus* other tetragonal bands for nanoparticles can be related to Ba vacancies in the BaTiO_3_^32^. Herein, the relative intensity of the peaks at 307 and 518 cm^−1^ has been calculated to evaluate the tetragonality of as-synthetized *x*Nd: BaTiO_3_ nanoparticles (the inset of Fig. 1f), which indicates a reduction trend of tetragonal distortions with the introduction of Nd ions.

### Defect chemistry of *x*Nd: BaTiO_3_ nanocrystals

**Figure 2:**
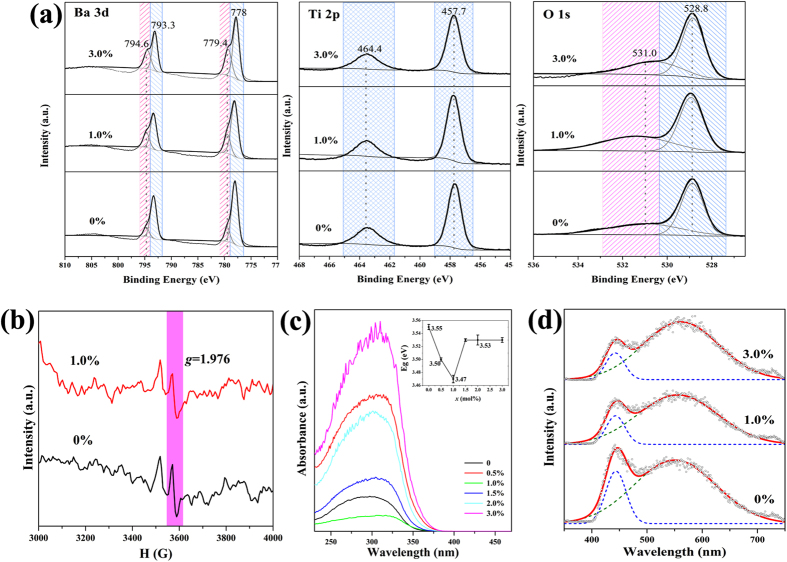
(**a**) XPS spectra for Ba *3d*, Ti *2p* and O *1* *s* of *x*Nd: BaTiO_3_ samples (*x* = 0, 1.0 and 3.0%): the black solid lines are the experimental data and the grey lines are the simulated curves; (**b**) Room-temperature EPR spectra of *x*Nd: BaTiO_3_ (*x* = 0, 1%); (**c**) UV-vis spectra in the absorbance mode for indicated *x*Nd: BaTiO_3_ samples; (**d**) Photoluminescence spectra of *x*Nd: BaTiO_3_ powders, where circles (grey) are the experimental data, the solid lines represent simulated spectra, and dashed lines are simulated individual emission peaks.

It is well-known that the properties of BaTiO_3_ are intimately related to the oxidation state of constituents. As-prepared *x*Nd: BaTiO_3_ nanoparticles were characterized by X-ray photoelectron spectroscopy (XPS) to determine the binding state and chemical environment of elements. Figure 2a shows photoemission spectra of Ba *3d*, Ti *2p*, and O *1 s* in *x*Nd: BaTiO_3_ nanoparticles. Peak deconvolution has been obtained by fitting the curves through the Gauss-Lorentz function. Peaks at 778 eV and 793.3 eV, respectively corresponding to the Ba *3d*_*5/2*_ and Ba *3d*_*3/2*_, are assigned to the perovskite structure of BaTiO_3_. It is worth pointing out that the higher shoulders at 779.4 eV and 794.6 eV are usually associated with Ba vacancy point defects^33^. Note that the peak intensity at both 779.4 eV and 794.6 eV increases with the increase in Nd concentration, suggesting that Ba vacancy would boost with the introduction of Nd ions. In order to reach charge balance, the substitution of Nd^3+^ for Ba^2+^ would either convert Ti^4+^ to Ti^3+^ or generate Ti vacancies 16,34,35. As reported in the literature^36^,^37^, the existence of Ti^3+^ ions would lead to the broad of the Ti *2p* peak and a lower binding energy shoulder belonging to 2p_3/2_ peak at ~456 eV. While such a feature has not been detected in our samples (see Fig. 2a), thus excluding the existence of Ti^3+^. Besides, the energy shift of Ti ions will extend over more than 4 eV in case of different coordination numbers^38^. Therefore, it can be concluded that the TiO_6_ octahedron is preserved. Figure 2a also presents the XPS spectra of O *1* *s* valence state, which shows one main peak corresponding to oxygen in BaTiO_3_ (528.8 eV) and a broad peak caused by chemisorbed species and/or oxygen vacancies (531.9 eV)^39^. Previously, Lewis Wasson *et al*.^40^ stated that the residual BaCO_3_ could hardly be detected by XPS, as the carbonate took the form of discrete particles rather than the continuous surface layer. Combining our previous FT-IR results^27^, it can be thus speculated that there exist chemisorbed OH^−^ ions.

Electron paramagnetic resonance (EPR) is a powerful technique to detect the presence of Ba and/or Ti vacancies^41^. Shown in Fig. 2b are the room-temperature EPR spectra of the pure BaTiO_3_ and 1%Nd: BaTiO_3_ nanopacrystals. Note that paramagnetic centers locate at *g* ~1.976 for both samples, implying the existence of Ba vacancies^42^. According to the previous report^43^, the formation of Ti vacancies would present a paramagnetic center at *g* = 2.004~2.005. Nevertheless, this signature of Ti vacancies has not been observed in our samples. From the XPS and EPR results, it can be concluded that the charge compensation mechanism primarily involves the formation of Ba vacancies induced by Nd substitution into A-site.

In general, any imperfection (vacancies, lattice defects, impurities/doping, and local bond distortion^44^) can yield density of states within the band gap of an insulator. Figure 2c shows the UV-vis absorption spectra of *x*Nd: BaTiO_3_ nanoparticles, with well-defined peaks and exponential tails. The Nd content dependent band gap (*E*_g_) is plotted as the inset, from which we can see the band gap (*E*_g_) initially decreases, then increases after *x* > 1% and eventually keeps constant at *x* > 1.5 mol%. This trend goes along with the Nd concentration dependent structural distortion observed in Raman spectra, where the structure of *x*Nd: BaTiO_3_ turns toward pseudo-cubic structure with the incorporation of Nd (see the inset of Fig. 1f). Probably, it is the structural transition that results in the constant band gap for *x *> 1.5 mol%.

To further characterize structural imperfection, we measured the photoluminescence (PL) emission spectroscopy at room temperature excited by laser with the wavelength of 355 nm. In general, the PL emission occurs when there is polarization within the structure and some localized states in the band gap, *e.g.* free exciton levels, self-trapped excitons and defects or impurity levels^45^. Figure 2d displays the emission spectra of as-prepared *x*Nd: BaTiO_3_ samples, where two peaks are observed at 446 nm and 554 nm, respectively. The former is considered to originate from a direct band excitation, intimately related to the distortion of TiO_6_^46^, as shown in blue dashed curves (blue wavelength region). The other peak is much broader (dashed green curves), which belongs to the yellow wavelength region according to Gaussian fit. This can be attributed to defects state within the band gap of the material^47^. The oxygen vacancies are considered as highly localized sensitive centers to trap electron from valance band and then the interaction of electron trapped with holes form self-trapped excitations. The radioactive recombination of the self-trapped excitations thus contributes to the emission of the yellow region. Compared to the low wavelength emission, this emission intensity increased gradually, indicating the oxygen vacancies defects increase with the incorporation of Nd ions.

Based on the above analysis, it can be concluded that when Nd is introduced into BaTiO_3_, Ba vacancies as well as oxygen vacancies would simultaneously form to compensate the charge balance, which can be described as Equation (1):


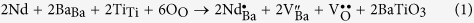


Previously, such an occupation mechanism has been taken into consideration in the study of RE-doped BaTiO_3_ ceramics. However, the existence of the Ba-vacancy induced by donor-doping mechanism(s) has never been demonstrated experimentally due to the mixtures of Ba_1-*y*_La_*y*_Ti_1-*y*/4_O_3_ and other Ti-rich phase(s) such as Ba_6_Ti_17_O_40_^48^. In our work, the formation of Ba-vacancy can be facilitated benefiting from the hydrothermal condition, where barium deficiencies are easily generated due to the introduction of protons^49^.

### Dielectric properties of *x*Nd: BaTiO_3_ ceramics

It is well-known that the partial substitution of Ba^2+^ by RE^3+^ would result in the decrease of Curie temperature *T*_C_ (corresponding to a cubic-to-tetragonal structure transition). In our work, the tetragonal structure observed at room temperature (Fig. 1d–f) indicates that the Curie temperatures *T*_C_ of *x*Nd: BaTiO_3_ nanocrystals still remain above room temperature, *i.e. T*_C_ > 300 K. In order to measure dielectric properties of our samples, *x*Nd: BaTiO_3_ ceramics were fabricated from as-synthesized *x*Nd: BaTiO_3_ nanocrystals by conventional pressureless sintering method at 1300 °C in air for 2 h.

**Figure 3:**
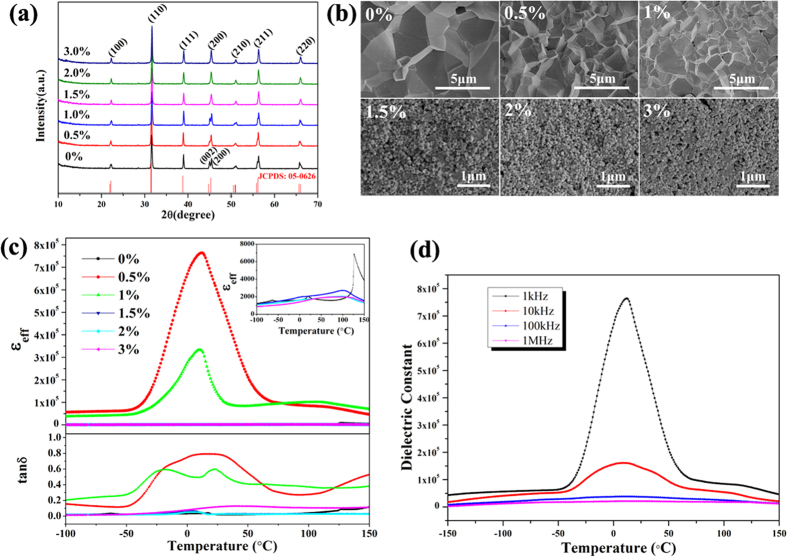
Properties of the Nd: BaTiO_3_ ceramics sintered at 1300 °C for 2 h. (**a**) XRD pattern of the *x*Nd: BaTiO_3_ samples; (**b**) SEM images of the *x*Nd: BaTiO_3_ ceramics; (**c**) Temperature dependence of the permittivity and dielectric loss for the *x*Nd: BaTiO_3_ ceramic samples measured at 1 kHz; (**d**) Permittivity versus temperature curves of 0.5%Nd: BaTiO_3_ ceramics at different frequencies.

As demonstrated in Fig. 3a, XRD patterns of *x*Nd: BaTiO_3_ ceramics corresponds to the tetragonal structure with lattice parameters *a* = *b* = 0.3994 nm and *c* = 0.4038 nm (JCPDS: No.05-0626). The peak splitting of (002)/(200) observed at 2*θ* ~46° for the samples with *x* ≤ 1 mol% suggests their tetragonal structures. At higher Nd contents (*x* > 1 mol%), the peak splitting at around 2*θ* ~46° cannot be recognized apparently. The results reveal that the introduction of Nd ions gives rise to the decrease in tetragonality, and induces the transition to pseudo-cubic structure. Cross-sectional FE-SEM images shown in Fig. 3b indicate that ceramic grain size decreases with the Nd content increasing. It’s observed that *x*Nd: BaTiO_3_ ceramics with *x* ≤ 1 mol% display compact microstructure, and their grain size is in the range of 1~5 μm. While the grain size of ceramic samples with *x* >1% is comparable to its counterpart nanocrystals, and loosen microstructures are observed in these ceramics.

Figure 3c shows the temperature dependent relative dielectric constant *ε*_eff_ and tangent loss tan*δ*. Two features are remarkable: (1) the pure BaTiO_3_ ceramics show a similar temperature dependence as conventional bulk ceramics with *T*_C_ ~125 °C, while the magnitude of *ε*_eff_ is about twofold higher; (2) the Nd introduction nonmonotonically enhances *ε*_eff_ and the colossal *ε*_eff_ ~7.6×10^5^ is observed in 0.5%Nd: BaTiO_3_ ceramics with tanδ ~0.8 at 12.2 °C.

In BT and rare-earth doped BT system, several explications have been proposed to explain the colossal dielectric constant, including internal barrier layer capacitance effect^50^, hopping polarization^19^, and electrode effect^51^. However, these ceramics either sintered in the non-oxidation atmosphere or sintered at a fast sintering rate with ultrafine grain size, which could result in the inner grain conductivity or the reduction of the Ti^4+^. Moreover, giant permittivity values were reported in hexagonal barium titanate (*h*-BaTiO_3_) single crystals^52^, and the high permittivity values ~100,000 of the oxygen deficient materials were explained by the presence of interfacial boundaries consisting of crystal defects. In addition, it is reported that in NaNbO_3_-doped BaTiO_3_ system^53^, colossal permittivity can be attributed to the high-energy electric state of Ba^+^ (or Ba^2+^ −e) by Ba^2+^ obtaining an electron, which can create electron hopping conduction and increase conductivity of the ceramics. These charged defects like Ba^+^ or Ba-vacancy are regarded to be responsible for the colossal permittivity^53^,^54^. However, neither the reduction of Ti^4+^ ions nor Ba^2+^ was observed in our work. In our viewpoint, the enhanced permittivity of *x*Nd: BaTiO_3_ ceramics (*x *≤ 1 mol%) should be on the one hand attributed to the compact microstructure, resulting from the highly-active *x*Nd: BaTiO_3_ nanoparticles. As we see in Fig. 3c, the permittivity of pure BaTiO_3_ ceramics is about twofold higher than that of conventional bulk ceramics. In combination with defect chemistry discussed above, the enhancement of the permittivity can be on the other hand ascribed to the space-charge polarization^55^, where the defects (*i.e.* Ba vacancies and O vacancies) induced by the dopants can act as additional dipoles. Since such a polarization cannot follow the alternating field at high frequency, the dielectric constant shows apparent decrease with the frequency arising. That is why the permittivity of the 0.5%Nd: BaTiO_3_ sample decreases dramatically with increasing frequency, where the maximum dielectric constant of ~160,000 and ~30,000 is observed at 10 kHz and higher frequencies (≥100 kHz), respectively (Fig. 3d).

In all, previous reports^19^,^56^ about the colossal permittivity in RE-doped BaTiO_3_ ceramics were almost achieved with the assistant of special atmosphere and/or sintering techniques. This work demonstrate that conventional pressureless sintering condition in air is adequate to obtain *x*Nd: BaTiO_3_ ceramics with colossal permittivity, as long as ceramic powders is of high sintering activity, which is a huge technique advantage in potential industrial production.

## Conclusion

A series of *x*Nd: BaTiO_3_ nanoparticles (*x* = 0 ~3.0 mol%) were successfully synthesized by a modified sol-hydrothermal method. It’s demonstrated that all samples exhibit tetragonal phase and their tetragonality gradually decreases with the increasing Nd content. Shoulder peaks at high binding energy reveal the possible coexistence of Ba and O vacancies, while the probable emergence of reductive Ti^3+^ is excluded. Furthermore, the existence of Ba and O vacancies is confirmed by EPR and PL analysis, respectively. It’s thereby proposed that the introduction of Nd ions into BaTiO_3_ nanocrystals induce the simultaneously formation of Ba and O vacancies due to the valence equilibrium. Controlled by the RE content, dielectric constant of ceramic samples initially increases and then falls. Among them, 0.5%Nd: BaTiO_3_ ceramics sintered from the nanopowders possess a room-temperature colossal permittivity (~760,000). The present work serves as a precedent and fundamental understanding of the crystal structure associated chemical defects of the RE-doped BaTiO_3_ nanopowders, and deliberate efforts are on-going to better understand the underlying mechanism for the room-temperature colossal permittivity.

## Methods

### Synthesis of *x*Nd: BaTiO_3_ nanoparticles

Nd-doped BaTiO_3_ nanocrystals (*x*Nd: BaTiO_3_, *x* = 0, 0.5, 1.0, 1.5, 2.0 and 3.0 mol%) were synthesized *via* a modified sol-hydrothermal method. To prepare TiO_2_ sol, Ti(OC_4_H_9_)_4_ and ethanol were fully blended with continuous stirring, and then a solution containing ethanol, HNO_3_ and deionized water was slowly added. The as-prepared TiO_2_ sol (10 ml) was then added to the solution of Ba(Ac)_2_ in 40 ml deionized water. Simultaneously, appropriate amount of Nd(NO_3_)_3_ solution was added to control the doping concentration. During the process, the alkalinity (2 mol/L) was regulated by a KOH solution. The resulting mixture was stirred for 20 min, and then the Teflon vessel was put into a stainless-steel autoclave. The sealed autoclave was heated to 200 °C for 16 h, and then cooled to room temperature naturally. After the synthesis, the precipitates were washed with deionized water and ethanol in sequence several times. The resulting precipitates were collected and frozen in a refrigerator, and then dried by a freeze drying devices (Alpha 1-2LD, Christ, Germany).

### Preparation of *x*Nd: BaTiO_3_ Ceramics

The as-prepared *x*Nd: BaTiO_3_ powders were mixed with 3 wt% PVA, pulverized using a mortar and pestle, and then pressed into pellets of 15 mm diameter under a uniaxial pressure of 8 MPa. After de-binding at 650 °C for 5 h, the green pellets were sintered at 1300 °C for 2 h in air by the conventional solid sintering method with a heating rate of 100 °C/h. Finally, the sintered ceramics were polished and coated with silver electrodes for electrical measurements.

### Characterization

Powder X-ray diffraction (XRD) was collected on a Bruker D8 Advance diffractometer operating at 40 kV and 40 mA using Cu Kα radiation (λ = 1.54178 Å) to determine the structure of obtained samples. Raman spectra were recorded in the wavenumber range of 100~1100 cm^−1^ using a Jobin Yvon T64000 (Jobin Yvon, France) excited by the laser with a wavelength of 800 nm. The elemental composition was obtained by inductively coupled plasma-optical spectroscopy (ICP-OES) on the Optima 5300DV (PE, USA). The morphology and microstructure were obtained using a Hitachi S-4800 (Hitachi, Japan) field emission scanning electron microscope (FE-SEM). High-resolution transmission electron microscopy (HR-TEM) and selected area electron diffraction (SAED) images were obtained with the use of Tecnai G2 F30 S-TWIN (FEI, USA) microscope operated at 200 kV. The valence states of elements were analyzed by X-ray photoelectron spectroscopy (XPS) using an Escalab 250Xi (ThermoFisher Scientific, USA). Electron paramagnetic resonance (EPR) measurements were performed at room temperature using a Bruker A300-10/12 spectrometer operating at 9.85 GHz. Photoluminescence (PL) spectra were recorded at room temperature by exciting the samples through a 355 nm He–Cd laser on a QM40-NIR (PTI, USA). UV-vis diffuse reflectance spectra were recorded on a UV-visible spectrophotometer TU-1901 (PGeneral Instrument Inc., China) at room temperature with BaSO_4_ as the reference and then converted into absorption spectra *via* Kubelka–Munk transformation. The dielectric properties of the samples were determined using the HP 4294 A (Hewlett-Packard, USA) impedance analyzer connected with a dc powder supply.

## Additional Information

**How to cite this article:** Sun, Q. *et al*. Crystalline Structure, Defect Chemistry and Room Temperature Colossal Permittivity of Nd-doped Barium Titanate. *Sci. Rep.* **7**, 42274; doi: 10.1038/srep42274 (2017).

**Publisher's note:** Springer Nature remains neutral with regard to jurisdictional claims in published maps and institutional affiliations.

## Supplementary Material

Supplementary Information

## Figures and Tables

**Table 1:** Nominal versus ICP-OES determined composition of *x*Nd: BaTiO_3_ nanocrystals.

Nominal composition	Analyzed composition	
	Ba/Ti	Nd (*x* mol)
BaTiO_3_	1.015	0%
0.5%Nd: BaTiO_3_	0.994	0.49%
1.0% Nd: BaTiO_3_	1.002	0.95%
1.5% Nd: BaTiO_3_	0.991	1.43%
