# Supplementary material for: Crystalline Structure, Defect Chemistry and Room Temperature Colossal Permittivity of Nd-doped Barium Titanate
Source: Sci Rep. 2017 Feb 13;7:42274. doi: 10.1038/srep42274 (PMC5304219; doi:10.1038/srep42274)
Supplement: Supplementary Information [file srep42274-s1.doc]

**Supplementary Information**

**Crystalline Structure, Defect Chemistry and Room-Temperature Colossal Permittivity of Nd-doped Barium Titanate**

Qiaomei Sun,^1, 2^ Qilin Gu,^1,2^ Kongjun Zhu,^*1^ Rongying Jin,^3^ Jinsong Liu,^2^ Jing Wang,^1^ Jinhao Qiu^1^

^1^State Key Laboratory of Mechanics and Control of Mechanical Structures, College of Aerospace Engineering, Nanjing University of Aeronautics and Astronautics, Nanjing 210016, China

^2^College of Materials Science and Technology, Nanjing University of Aeronautics and Astronautics, Nanjing 210016, China

^3^Department of Physics and Astronomy, Louisiana State University, Baton Rouge, LA 70803, USA

*Corresponding Author, E-mail address: kjzhu@nuaa.edu.cn

**Fig. S1** shows the microstructure of the as-prepared 1%Nd: BaTiO_3_ products. Well-dispersed nanoparticles with similar morphologies can be clearly observed from the FE-SEM image (Fig. S1a). According to the selected area electron diffraction (SAED) (the inset images in Fig. S1c and Fig. S1e), both the spherical and square-shaped particles exhibit single crystal diffraction spots. The HR-TEM pattern of the single particle marked by yellow box in Fig. S1c is presented in Fig. S1d, and the orderly arranged atoms can be seen along the [111] crystal orientation. The calculated lattice spacing is 0.283 nm and 0.281 nm, corresponding to the (011)/(101) and the (110) planes, respectively. Moreover, the atomic arrangements seen from the [001] zone axis (Fig. S1f) indicate that the interplanar spacing of the (100)/(010) planes remains 0.397 nm, which represents the obtained crystals take a general cell parameter (a = b = 0.397 nm, c = 0.403 nm). The derived unit cell parameters demonstrate the particles possess tetragonal structures, which are consistent with the XRD and Raman results.

The overall morphologies of the 3%Nd: BaTiO_3_ are analyzed, as shown in Fig. S2. Some rod-like particles among the spherical particles can be seen in the SEM images (Fig. S2a), while there is no obvious distinguish in the BT particles, as determined by the HR-TEM results shown in Fig. S2d and Fig. S2e. In combination with the XRD results, it is presumed that the nanorods pertain to the Nd(OH)_3_. Thus, EDXS analysis is implemented to dissect the components of the nanorods. Qualitative element analysis reveals that the main elements of rod-like particle are Nd and O while minor Ba or Ti is detected. The existences of Ba and Ti may be resulted from the diffusion of the Ba and Ti ions in the solution, which is similar to the Kirkendall diffuse mechanism reported by Meng-Fang Lin *et al*^1^. From these observations, it can be concluded that, ignoring the nanorods emerged in the highly Nd doped samples, no significant changes in the microstructure of the BT powders are observed with the Nd incorporation.


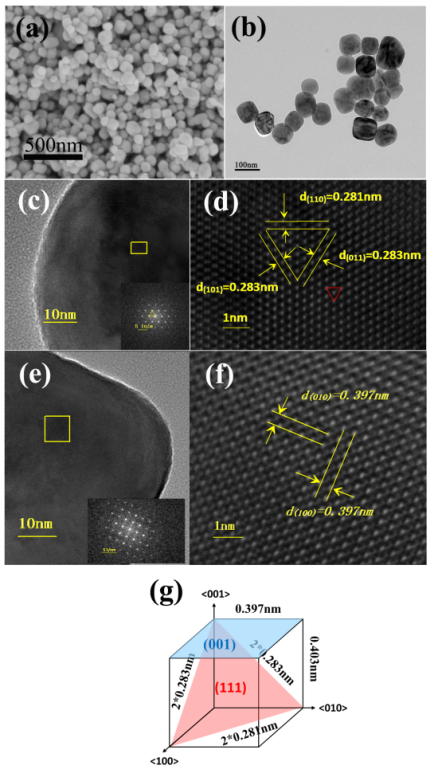


Figure S1: (a) SEM and (b) TEM images of 1%Nd: BaTiO_3_ nanoparticles; (c) Magnified TEM images of an individual spherical particle and the inset is the SAED pattern and (d) the HR-TEM images of the selected area labeled by the yellow square in (c); (e) Magnified TEM images of a square-shaped particle and the inset is the fast Fourier transform SAED pattern and (f) the HR-TEM images of the selected area labeled by the yellow square in (e); (g) The schematic illustration of the unit cell.


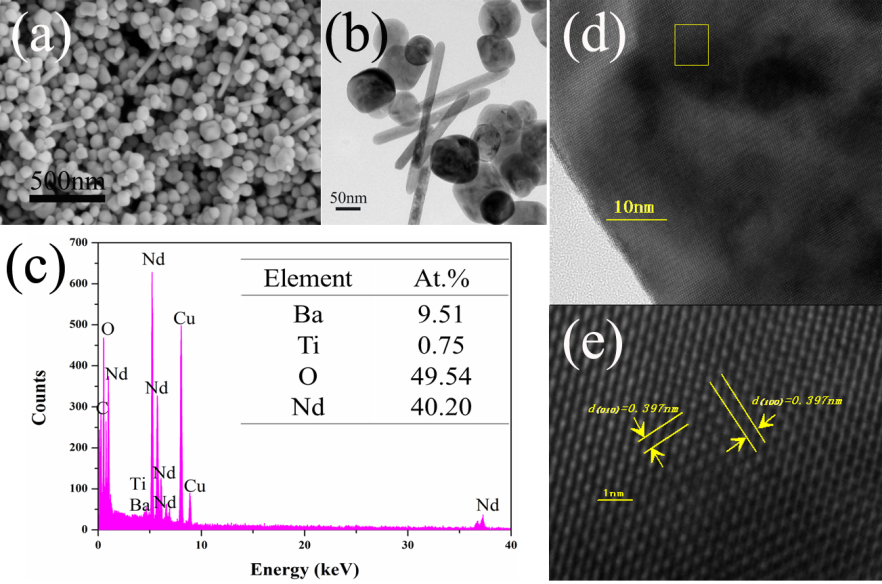


Figure S2: (a) SEM and (b) TEM images of 3%Nd: BaTiO_3_ nanoparticles; (c) EDX and element analysis of the nanorods; (d) Magnified TEM images of individual particle and (d) the HR-TEM images of the selected area labeled by the yellow square in (d).

**References**

1. Lin, M. F., Thakur, V. K., Tan, E. J. & Lee, P. S. Dopant induced hollow BaTiO_3_ nanostructures for application in high performance capacitors. *J. Mater. Chem.* **21**, 16500 (2011).
